# A Curated Cell Life Imaging Dataset of Immune-enriched Pancreatic Cancer Organoids with Pre-trained AI Models

**DOI:** 10.1038/s41597-024-03631-3

**Published:** 2024-07-24

**Authors:** Ajinkya Kulkarni, Nathalia Ferreira, Riccardo Scodellaro, Dolma Choezom, Frauke Alves

**Affiliations:** 1 Translational Molecular Imaging, Max Planck Institute for Multidisciplinary Sciences, Hermann-Rein-Straße 3, 37075 Göttingen, Germany; 2 Department of Haematology and Medical Oncology, University Medical Center Göttingen, Robert-Koch-Straße 40, 37075 Göttingen, Germany; 3 Department of General, Visceral and Pediatric Surgery, University Medical Center Göttingen, Robert-Koch-Straße 40, 37075 Göttingen, Germany; 4 Institute for Diagnostic and Interventional Radiology, University Medical Center Göttingen, Robert-Koch-Straße 40, 37075 Göttingen, Germany

**Keywords:** Preclinical research, Drug development

## Abstract

Tumor organoids are three-dimensional *in vitro* models which can recapitulate the complex mutational landscape and tissue architecture observed in cancer patients, providing a realistic model for testing novel therapies, including immunotherapies. A significant challenge in organoid research in oncology lies in developing efficient and reliable methods for segmenting organoid images, quantifying organoid growth, regression and response to treatments, as well as predicting the behavior of organoid systems. Up to now, a curated dataset of organoids co-cultured with immune cells is not available. To address this gap, we present a new public dataset, comprising both phase-contrast images of murine and patient-derived tumor organoids of one of the deadliest cancer types, the Pancreatic Ductal Adenocarcinoma, co-cultured with immune cells, and state-of-the-art algorithms for object detection and segmentation. Our dataset, *OrganoIDNetData*, encompassing 180 images with 33906 organoids, can be a potential common benchmark for different organoids segmentation protocols, moving beyond the current practice of training and testing these algorithms on isolated datasets.

## Background & Summary

Pancreatic Ductal Adenocarcinoma (PDAC) is the most prevalent neoplastic disease of the pancreas, accounting for over 90% of all pancreatic malignancies^1^. Characterized by its aggressive nature PDAC patients with metastasis have a median life expectancy <1 year. Such dismal prognosis can be traced back to late detection, tumor heterogeneity, intrinsic chemoresistance and failure of conventional therapeutic approaches. Moreover, the chemotherapeutic strategies as cornerstone of treatment often lead to increased side-effects and toxicity amongst the PDAC patients, accompanied with only a slight increase in survival rates^2^. Therefore, especially PDAC poses a formidable challenge in the field of oncology. Offering a distinct approach, immunotherapy has transformed cancer treatment strategies, demonstrating fewer side effects and reduced tumor resistance compared to conventional chemotherapy^3^. However, applying immunotherapeutic strategies to PDAC patients still faces obstacles. PDAC is an inherently immune-cold tumor and employs various strategies to counteract the effectiveness of immunotherapy, such as a dense stroma that i) hinders the infiltration of T-cells into the PDAC tumor microenvironment, ii) promotes chemoresistance and immune escape, and iii) produces cytokines that support the growth and survival of the tumor cells^4^. Additionally, the translation of immunotherapy into clinical applications is impeded by the inadequacy of effective pre-clinical models.

Although traditional animal models have been extensively utilized, they fall short in replicating the unique PDAC tumor microenvironment (TME), immune evasion mechanisms, and the distinctive genetic landscape of PDAC^5^. Acting as a bridge between two-dimensional cell lines and advanced patient-derived xenograft (PDX) models, organoids serve as a three-dimensional *in vitro* model capable of faithfully reproducing the complex mutational landscape observed in PDAC patients. Furthermore, patient-derived organoids (PDOs) further provide a platform to mimic the unique genetic characteristics of PDAC patients, advancing as a pre-clinical model that facilitates personalized treatment options in clinical practice. Organoids exhibit characteristics such as self-renewal and self-organization into mini-organ structures that closely resemble the original tissue architecture^6^. Despite the significant strides PDAC organoids bring to pre-clinical testing of novel therapies, they fall short in maintaining the immune system compartment of the PDAC-TME, posing a challenge for evaluating new immunotherapeutic strategies^7^ in this model.

To address the absence of the immune system compartment, researchers have been refining protocols to create an optimal environment that facilitates the co-culture of organoids with the immune system^8^. This endeavor has been instrumental in testing immunotherapies, including antibody-based immune checkpoints, leading to novel discoveries in the immunotherapeutic field^9^. The practical evaluation of treatment efficacy in organoids involves the use of Cell-Titer Glo as an endpoint assay, allowing the quantification of metabolic active cells and assessment of organoid viability^10^. Nevertheless, live cell imaging was discovered as a breakthrough technology to evaluate live response of organoids, thanks to its capability to monitor the real time reaction of organoids towards the therapy during every steps of the treatment^11–13^.

Up to now, several algorithms have emerged to analyze the response of organoids during live cell imaging to specific treatments based on physical measurements of organoid properties, such as mean area, organoid eccentricity and total organoid count. These algorithms enable the continuous assessment of organoid reactions over time without disrupting the organoid system^14–18^. Nevertheless, each algorithm is evaluated on its proper dataset, in optimal conditions, not allowing a valid comparison among the different performances. Moreover, all of these datasets report organoids in a relatively simple environment, where co-culture with immune cells has not been considered.

Here, we propose OrganoIDNetData, a public dataset of phase-contrast images of human and murine PDAC organoids, co-cultured with immune cells. This dataset provides a curated common benchmark among different segmentation protocols, checking their performances in a more complex environment, incorporating pre-activated peripheral mononuclear cells (PBMCs) into the system. Together with the images, the dataset also provides the scripts to analyze the data with two different state-of-the-art algorithms for image segmentation and object detection, Cellpose^19^ and StarDist^20^.

## Methods

### Patient tissue processing and organoid cultures

All samples were prepared as detailed extensively in previous literature^21^. Here, we provide a brief overview of the procedures we followed.

#### Human Cultures

All specimens of Pancreatic Ductal Adenocarcinoma (PDAC) tumors from human patients, used in the generation of PDAC Patient Derived Organoids (PDOs), were sourced during surgical intervention from individuals participating in the Molecular Pancreas Program (MolPAC) at the University Medical Center of Göttingen (UMG). This was done in accordance with the approval granted by local regulatory authorities (Ethics Committee of the UMG), as indicated by approval references 11/5/17, 22/8/21Ü, and 2/4/19. In the case of human Peripheral Blood Mononuclear Cells (PBMCs), peripheral blood was obtained from anonymous healthy donors with approval from the Ethics Committee of the UMG (ETHIC approval 29/07/23). The participants consented to the open publication of the anonymized data obtained from these samples.

#### Murine Cultures

To generate murine PDAC tumors, a quantity of 5 × 10^5^ KPC cells was orthotopically implanted into the pancreatic head of C57BL/6 J mice, following previously established procedures^22^. Murine peripheral blood was collected from euthanized healthy C57BL/6J mice through cardiac puncture. All experiments were conducted with approval from the administration of Lower Saxony (approval number G20.3527) in accordance with current German laws governing animal experimentation.

#### Medium Preparation

- Digestion medium: Composition per 100 *ml* included 12 *mg* Collagenase type I (Sigma), 12 *mg* Dispase II (Sigma), and 1 *ml* of 10% FCS (Gibco) in 99 *ml* of DMEM (Gibco). Human Organoid Growth Medium (HOGM): 50 *μl* of HOGM consisted of 25 *μl* A83-01 (1*mM*, Tocris), 50 *μl* Human Epidermal Growth Factor (hEGF; 500 *μg*/*ml*, Invitrogen), 50 *μl* human Fibroblast Growth Factor-10 (hFGF-10; 100 *mg*/*ml*, Peprotech), 50 *μl* Gastrin I (100 *μM*, Sigma), 125 *μl* N-acetylcysteine (500 *mM*, Sigma), 500 *μl* Nicotinamide (1 *M*, Sigma), 1 *ml* B-27 supplement (50x, Gibco), 100 *μl* Primocin (50 *mg*/*ml*, InvivoGen), with additional components diluted in 19 *ml* of organoid splitting medium (1*x* Glutamax, 1*x* HEPES, 1 *ml* 1*x* Primocin, 30% Bovine Serum Albumin (BSA) diluted in Advanced DMEM/F12 medium (AdDMEM/F12, Gibco). For initial seeding, splitting, or thawing, 1:1000 Rho Kinase Inhibitor (Sigma) was added.
- Murine Organoid Growth Medium (MOGM): 50 *ml* of MOGM contained 5 *μl* murine Epidermal Growth Factor (mEGF; 500 *μg*/*ml*l, Invitrogen), 50 *μl* murine Fibroblast Growth Factor-10 (mFGF-10; 100 *μg*/*ml*, Peprotech), 5 *μl* Gastrin I (100 *μM*, Sigma), 125 *μl* N-acetylcysteine (500 *mM*, Sigma), 500 *μl* Nicotinamide (1 *M*, Sigma), 1 *ml* B-27 supplement (50x, Gibco), 5 *ml* R-spondin, and 5 *ml* of Noggin-conditioned media diluted in 38.3 *ml* of organoid splitting medium (1*x* Glutamax, 1*x* HEPES, 1% Penicillin-Streptomycin diluted in AdDMEM/F12). Similar to HOGM, 1:1000 Rho Kinase Inhibitor (Sigma) was added for initial seeding, splitting, or thawing.
- Wnt3a-, R-Spondin- and Noggin-conditioned media: Wnt3a-, R-Spondin-, and Noggin-conditioned media were prepared as detailed in previous literature^23,24^. Conditioning involved culturing specific cell lines (L-Wnt3A for Wnt3a, 293T-HA-Rspol-Fc for R-Spondin, and HEK293-mNoggin-Fc for Noggin) followed by collection, centrifugation, pooling, and filter sterilization before storage at −20 °C.
- Organoid passaging medium (OPM): 500 *ml* of OPM included 5 *ml* 100x Glutamax, 5 *ml* 1 *M* HEPES, and 1% Penicillin-Streptomycin in 500 *ml* AdDMEM/F12.
- PBMCs culture medium: RPMI medium supplemented with 10% FCS, 50 *μMβ*-mercaptoethanol, and 1% Penicillin-Streptomycin.

#### PDAC organoid establishment and culturing

PDAC organoids, obtained from either murine or human PDAC tumors, were generated following established organoid cell culture procedures outlined^21,25^. In summary, cells were isolated from freshly excised PDAC tumor samples and exposed to a digestion medium. Subsequently, the cells were suspended in 50 *μl* of Growth Factor Reduced (GFR) Matrigel (Corning) to form a dome within a preheated 24-well plate. Upon solidification of the dome, the respective organoid growth medium for human and murine PDAC organoids was applied to the top. Human and murine organoid formation was observed by live cell imaging (see the “Live cell imaging protocol” subsection for further information) after 3 days of culturing at 37 °C in a humidified atmosphere with 5% *CO*_2_. The organoids were used for experimentation after undergoing a minimum of 3 passages.

#### PDAC organoids co-cultures with PBMCs

Following the 3-day formation of PDAC organoids, a co-culture with PBMCs was initiated. Simultaneously, human and murine PBMCs underwent pre-activation through culturing in PBMCs culture medium supplemented with ImmunoCultTM Human CD3/CD28 T cell Activator (StemCell Technologies) and suspension in LymphoGrow II medium (Cytogen), respectively. After an overnight pre-activation phase, co-cultures were established at a ratio of 10^4^ organoids to 10^5^ pre-activated PBMCs. The medium, comprising 50% MOGM or HOGM with 50% PBMC medium, was then added.

### Imaging protocol and dataset preparation

#### Live cell imaging protocol

The growth and viability of PDAC organoids and PBMCs organoid co-cultures were monitored with the live-cell imaging system IncucyteR S3 (Sartorius, Germany). The Incucyte S3 system from Sartorius proves to be a valuable asset for observing the time-lapse behavior of organoids in response to a particular treatment. This in-incubator microscope system offers label-free, automated acquisition of organoids within a physiologically stable environment favorable to organoid growth. 180 Phase-contrast fields of view of a mixture of sizes (1408 × 1040 pixels & 1280 × 852 pixels) were acquired every 4 hours up to 100 hours using the *Organoid* mode. The 4*x* objective provided a resolution of 2.82 *μm* per pixel.

#### Dataset Creation

The process of generating the proposed grayscale dataset used for training the model commenced with the extraction of the acquired field of views, as described by the live cell imaging protocol. The dataset was then meticulously verified for integrity and completeness. To achieve this purpose, we used several custom-made functions, available in the dedicated repository^26^, maintaining the same nomenclature as used in this description. The “*count_image_names_and_check_masks*” function from the “*Prepare_Dataset_Modules.py*” script ensures that each field of view has a corresponding mask (generated by experts by manually annotating and segmenting the organoids) and identifies the quantity of fields of view belonging to human patients or murine cultures, by checking their prefixes “*human_*” and “*mouse_*”.

Following this, the “*extract_patches*” function was employed to segment the larger RGB images and masks into smaller grayscale patches, respectively converted into 8-bit and 16-bit, and stored in TIFF format. The “*extract_patches*” function is also available as a separate package at the dedicated GitHub public repository^26^. The patching was guided by specific parameters, including the patch size of size 800 × 800 pixels, overlap percentage of 200 pixels in horizontal and vertical directions. A minimum threshold of 4 labels per patch was incorporated, ensuring that each patch contained sufficient data for effective training. These parameters can be personalized by the user by modifying the input values in the provided script.

Subsequent to patch creation, a representative subset of these patches was selected and visually inspected to confirm their suitability for training. This crucial step validated the quality and applicability of the patches. Following this, the dataset underwent a division into training, validation and testing sets based on a pre-determined ratio (75% for training and 20% for validation and 5% for testing). This split was carefully executed to ensure a balanced distribution of data. This is followed by the augmentation phase, using the augment function from “*Prepare_Dataset_Modules.py*” to each image in the training set. This function systematically applies a series of random transformations, such as flipping, rotation, translation, zooming, and adjustments in brightness and contrast to introduce variability in the dataset. Each image was subjected to a specified number of augmentations (as determined by the “*n_augmentations*” variable, in this study fixed at 2, due to our limited computational resources), thereby enriching the dataset with diverse representations of the original data. If more augmentations are supported by the user’s hardware, the code can be run with a different value of the parameter “*n_augmentations*” to increase the cardinality of the augmented dataset. To provide a preliminary indication for users in choosing an appropriate number of augmentations, we tested various data augmentations on the OrganoIDNetData dataset. Due to our computational limitations, we trained Cellpose and Stardist using a random selection of 25% of the dataset images. Our findings indicate that n = 6 provided better results in terms of object-wise segmentation accuracy, if compared to n = 2. We advise against selecting n ≥ 10, as overfitting was observed with both Cellpose and Stardist approaches at n = 10.

The final stages of the process included comprehensive checks for data sanity and validation using the “*check_data_sanity*” and the “*validate_and_count_images*” functions. These functions ensure that the dataset maintains its structural integrity, adheres to the expected data types and value ranges (0-255 for the 8-bit format patches, and 0-65535 for the 16-bit format masks), and provide a transparent overview of its composition, including the count of human and mouse prefixed images and masks. Finally, the “*count_organoid_number_and_report*” function automatically counts and reports the number of organoids in the masks folder. It calculates both the total and average number of organoids across all the masks, ignoring the background.

#### Manual image segmentation protocol

Each image of the dataset was manually segmented by two different annotators, who are researchers with extensive expertise in the organoids field. The annotations were performed independently, ensuring that no bias was introduced through mutual consultation. Both annotators used the Labkit tool available in Fiji software^27^. To provide the ground truths, in the presence of discrepancies between the two annotations, the two experts decided a common segmentation after consultation.

#### Automated image segmentation protocols

To validate the dataset and demonstrate that it can be a potential challenging benchmark to compare different segmentation and object detection protocols, we analyzed it by training two state-of-the-art algorithms: Cellpose^19^ and StarDist^20^. Cellpose is a deep learning-based method for cellular segmentation of in microscopy images. Cellpose works by using a convolutional neural network (CNN) to identify and segment cells in images. Particularly suitable for fluorescence and phase-contrast images, Cellpose is characterized by a great versatility, since the CNN is trained on a dataset including images of cells of different types, sizes, and conditions. Characterized by a simpler architecture than Cellpose, StarDist is a deep-learning method primarily designed for segmenting cells *in vitro* and *in vivo*. It also relies on CNNs, and uses a star-convex polygon approach to segment objects by defining convex polygons around them. StarDist is particularly designed for addressing challenges in segmenting elongated and branched structures in microscopy images. Both the algorithms were trained for 1000 epochs and the weights were saved for future inference. Additionally, all other parameters which are used to train both models are also available in the GitHub repository^26^ as a part of the source codes. Specifically, both the models were completely re-trained. For training the model using Cellpose, the “*initial_diameter*” parameter was kept to 0, with a batch size of 4 and the “*flow_threshold*” parameter set to 0.4. For training the model with StarDist, the “*n_rays*” parameter was kept at 32, with a grid size of (2, 2).

## Data Records

**Fig. 1:**
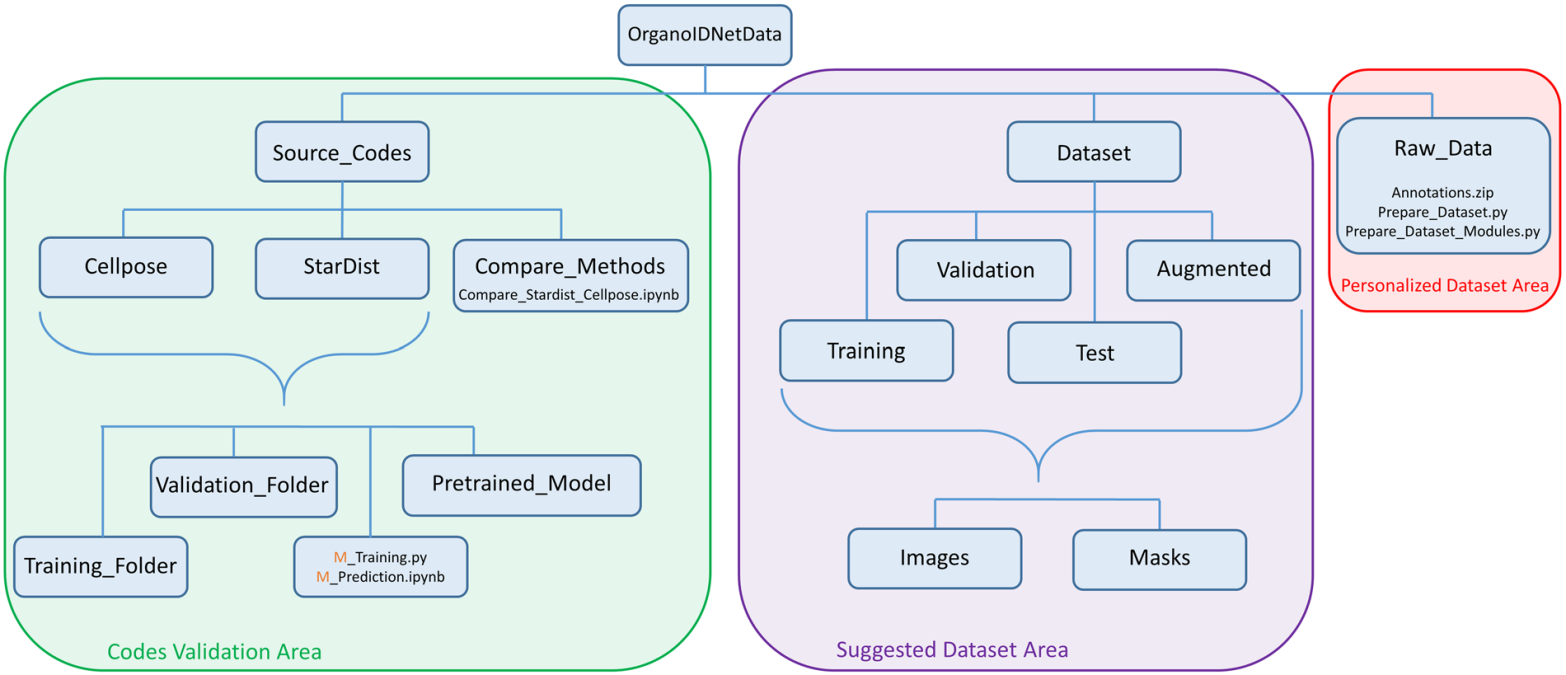
OrganoIDNetData file structure. Three main subfolders are contained in the OrganoIDNetData repository, recognizing three different areas of the directory. The “*Source_Codes*” subfolder stores all the python codes needed to reproduce the technical validation performed by using Cellpose and StarDist algorithms. The “*Raw_Data*” subfolder houses all the raw field of views, experimentally acquired to create the OrganoIDNet dataset, together with the python scripts that the user can modify and create its personalised version of the dataset. The “*Data*” subfolder stores the complete dataset presented in this article, organised in training, augmented, validation and test datasets.

The OrganoIDNetData dataset is public and can be freely downloaded from the corresponding Zenodo repository^26^, with the corresponding source codes. It consists of systematically organized grayscale microscopy images of PDAC organoids, co-cultured with immune cells, in TIFF format, and corresponding segmented mask files, aimed at supporting organoid research. For visualization, we suggest opening both images and masks with ImageJ or Fiji softwares. For a clear understanding of the file structure, we provided a diagram in Fig. 1, which depicts the three subfolders contained in the OrganoIDNetData main directory.

The “*Source_Codes*” subfolder houses the Python codes used to perform the model training and conduct the technical validations of the manuscript. For both algorithms, Cellpose and StarDist, users can replicate the results by running these Python scripts. The “*M_Training.py*” script provides the training steps for each method, where M describes the approach (Cellpose or StarDist), while “*M_Prediction.ipynb*” can be used to analyze test data, once the method is trained. The data required for this process is readily available in the “*Training_Folder*” and “*Validation_Folder*” subfolders of the corresponding method. Alternatively, users can upload their own images to these subfolders and run the Cellpose and StarDist codes using their own data. However, both architectures, already trained with the proposed dataset, are stored in the “*Pretrained_Model*” subfolder. Additionally, the “*Compare_Methods*” folder houses the scripts necessary to obtain the quantitative metrics able to provide a comparison among different training methods. At the moment, the scripts are limited to the comparison of Cellpose and StarDist.

The “*Raw_Data*” folder houses a compressed archive, called Annotations.zip containing all the raw fields of view used to create the dataset. Specifically, organoids derived from four different patients, and co-cultured with immune cells, four human organoid cultures were established from primary tumors obtained from four independent PDAC patients. Additionally, two murine organoid cultures were derived from primary tumors obtained from two separate PDAC bearing mice. Out of the 180 fields of view, 94 were derived from human cultures, while the remaining 86 were from murine cultures. Moreover, this subfolder stores two Python scripts: “*Prepare_Dataset.py*” and “*Prepare_Dataset_Modules.py*”. These scripts are pre-configured to process the raw field of views and generate the dataset presented in this article. The scripts are commented to enhance the versatility of the dataset by letting the user create its personalised version of the dataset. However, the code can be modified to create new datasets with different image dimensions.

The “*Dataset*” folder also stores the complete dataset presented in this article, obtained by all the raw field of views. The images are organized into distinct subfolders. The “*Training*” subfolder contains 713 images (364 from human cultures, 349 from murine cultures) of 800 × 800 pixels, which are used to train the network. Conversely, the “*Validation*” subfolder holds 179 (98 from human cultures, 81 from murine cultures) images, which are used to optimize the network’s performance and parameters during the training. The “*Augmented*” subfolder contains 1426 augmented images generated by applying data augmentation on the training set. These augmented images were added to the standard training set to expose the network to a wider variety of image representations and used during the technical validations presented in this article. The “*Test*” subfolder contains 10 entire fields of view (5 from human organoid co-cultures, 5 from murine organoid co-cultures), not splitted in patches, that can be used to evaluate the network’s performance on unseen data. Within each of these subfolders, images are further organized into two subfolders: the “*Images*” subfolder contains the actual images comprising our dataset, while the “*Masks*” subfolder stores the corresponding masks for each image, which were obtained by following the manual annotation protocol already described in the corresponding subsection.

**Fig. 2:**
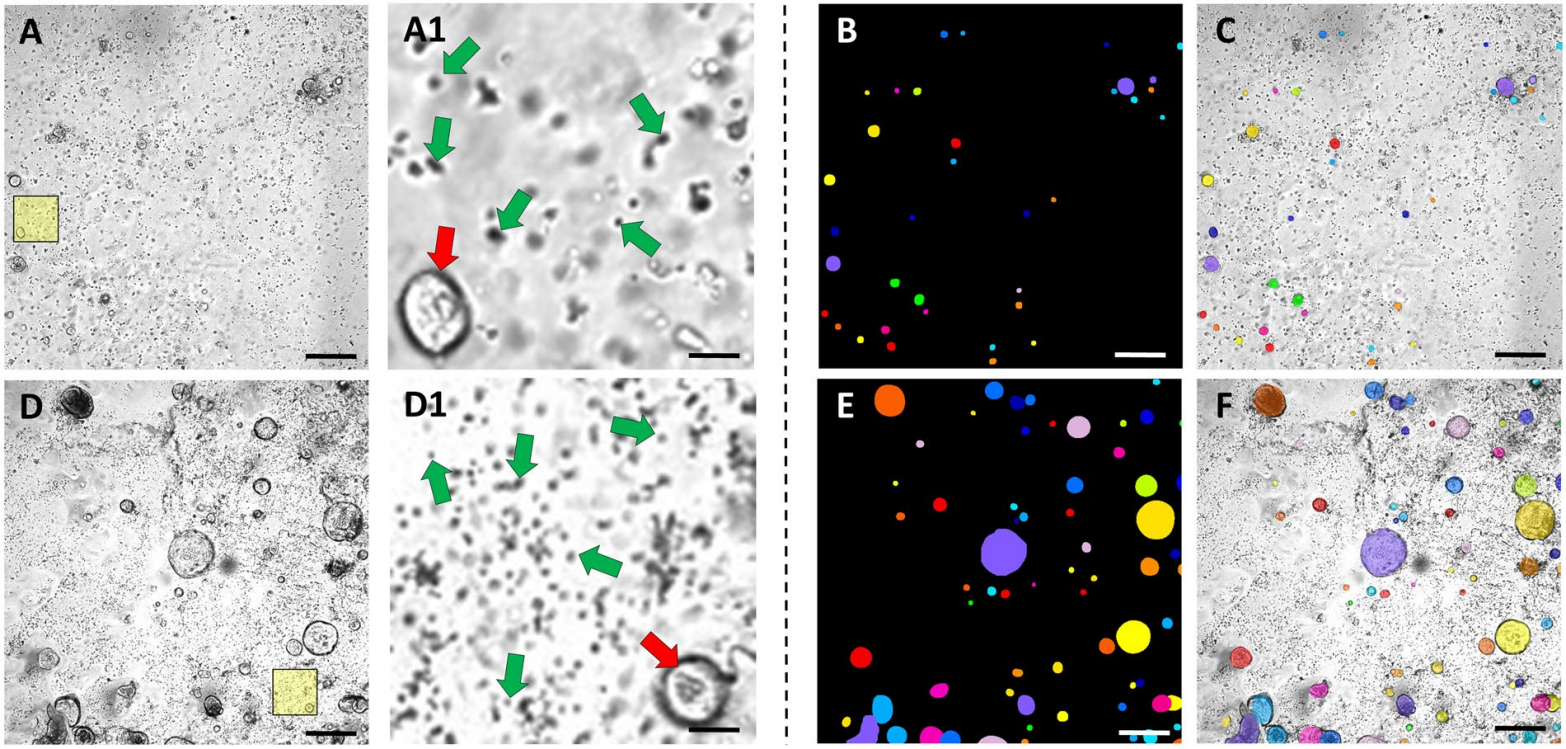
Representative images and corresponding masks of the OrganoIDNetData dataset. (**A**) and (**D**) report two dataset images, “*Human_1_024h_patch3.tif*” and “*Mouse_1_042h_patch0.tif*”, respectively. The two yellow squares highlight the zoomed-in regions of interest shown in (A1) and (D1). In each region of interest, a single organoid is indicated by a red arrow, while five immune cells, randomly selected, are highlighted by green arrows. (**B**) and (**E**), whose files are named “*Human_1_024h_patch3_mask.tif*” and “*Mouse_1_042h_patch0_mask.tif*”, show the corresponding manual segmentations for images (**A**) and (**D**), respectively. (**C**) and (**F**) depict the overlay between the original images of the dataset and the masks to better highlight the quality of the annotation. Scale bars: 115 *μm* for images from (**A**) to (**F**), 15 *μm* for images (A1) and (D1).

Both images and masks are saved in TIFF format and named using a standard convention that encodes their characteristics. The file name structure follows the pattern “*type_X_Wh_patchY_augZ.tif*”, where type *X* specifies the sample origin, either human or mouse. *X* is a unique identifier for the sample, *W* recognizes the time step of the measurement, expressed in hours, and patch identifies a distinct 800 × 800 pixels Y patch extracted from the same sample. The presence of the “*_augZ*” string is optional and indicates that the image was obtained through data augmentation, where the *Z* number distinguishes augmented data generated from the same image with different augmentation parameters. The mask filename follows the same pattern as the corresponding image filename, but with the final string “*_mask*” appended. For instance, the file “*Human_1_016h_patch4.tif*” recognizes the fourth patch extracted by the field of view acquired for the first human sample after 16 hours from the beginning of the live cell imaging acquisition. Conversely, “*Human_1_016h_patch4_aug2.tif*” is associated to the second augmented image generated by the original one, while their corresponding masks are “*Human_1_016h_patch4_mask.tif*” and “*Human_1_016h_patch4_aug2_mask.tif*”. When an entire field of view is provided, as in the case of the “*Raw_Data*” and “*Test*” subfolders, the string “*_patchY*” is not present in the name of the files. Moreover, each field of view we propose as part of the test dataset, and present in the “*Test*” subfolder, is characterized by “*Test_*” at the beginning of their name, following the structure “*Test_type_X_Wh.tif*”. Figure 2 displays two exemplary images (and their respective masks) from the dataset: one featuring organoids derived from a human patient and the other illustrating organoids from a murine sample. Some immune cells present in these two images are highlighted by manual annotation.

## Technical Validation

Here, we present some proof-of-concept demonstrations showcasing the functionalities available to users with OrganoIDNetData. Given our proposition of the dataset as a potential common and standardized benchmark for various segmentation protocols, we conducted a comprehensive analysis of the OrganoIDNetData dataset. Firstly, we assessed the quality of the manual annotations through an inter-operator agreement analysis. We then trained two state-of-the-art algorithms for automated segmentation, StarDist and Cellpose, using the OrganoIDNetData training dataset and tested them on the OrganoIDNetData test dataset to compare their performance. We conducted a detailed error analysis for both algorithms, examining the features of incorrectly segmented PDAC organoids to identify the most challenging morphological aspects for segmentation that our dataset provides. A similar analysis was performed on time-lapsed images from the proposed dataset to demonstrate if the ability of segmentation protocols to track various morphological features of organoids over time can be tested with OrganoIDNetData. Furthermore, we evaluated the dataset biological variability in terms of the morphology of the human PDAC organoids co-cultured with immune cells derived from the four different patients by independently analyzing the distributions of specific features (area, jaggedness, compactness, and eccentricity). We assessed whether this variability could affect the training phase’s convergence and potential overfitting, increasing the challenge of an accurate segmentation of the OrganoIDNetData images. Finally, we demonstrated the higher complexity of our immune cell-enriched dataset compared to state-of-the-art datasets lacking immune cells. This was achieved by performing an in silico ablation of immune cells from OrganoIDNetData, followed by training StarDist and Cellpose with the modified dataset. A comparative morphological analysis of human PDAC organoid co-cultured with and without immune cells was also conducted to further evaluate the complexity of our dataset in terms of organoids shape. All analysis presented here was performed on a desktop workstation with an AMD Ryzen 9 5900X 12-Core Processor with 128 GB RAM, and an NVIDIA GeForce RTX 3080 GPU with dedicated 12 GB RAM.

### Manual annotation evaluation

**Fig. 3:**
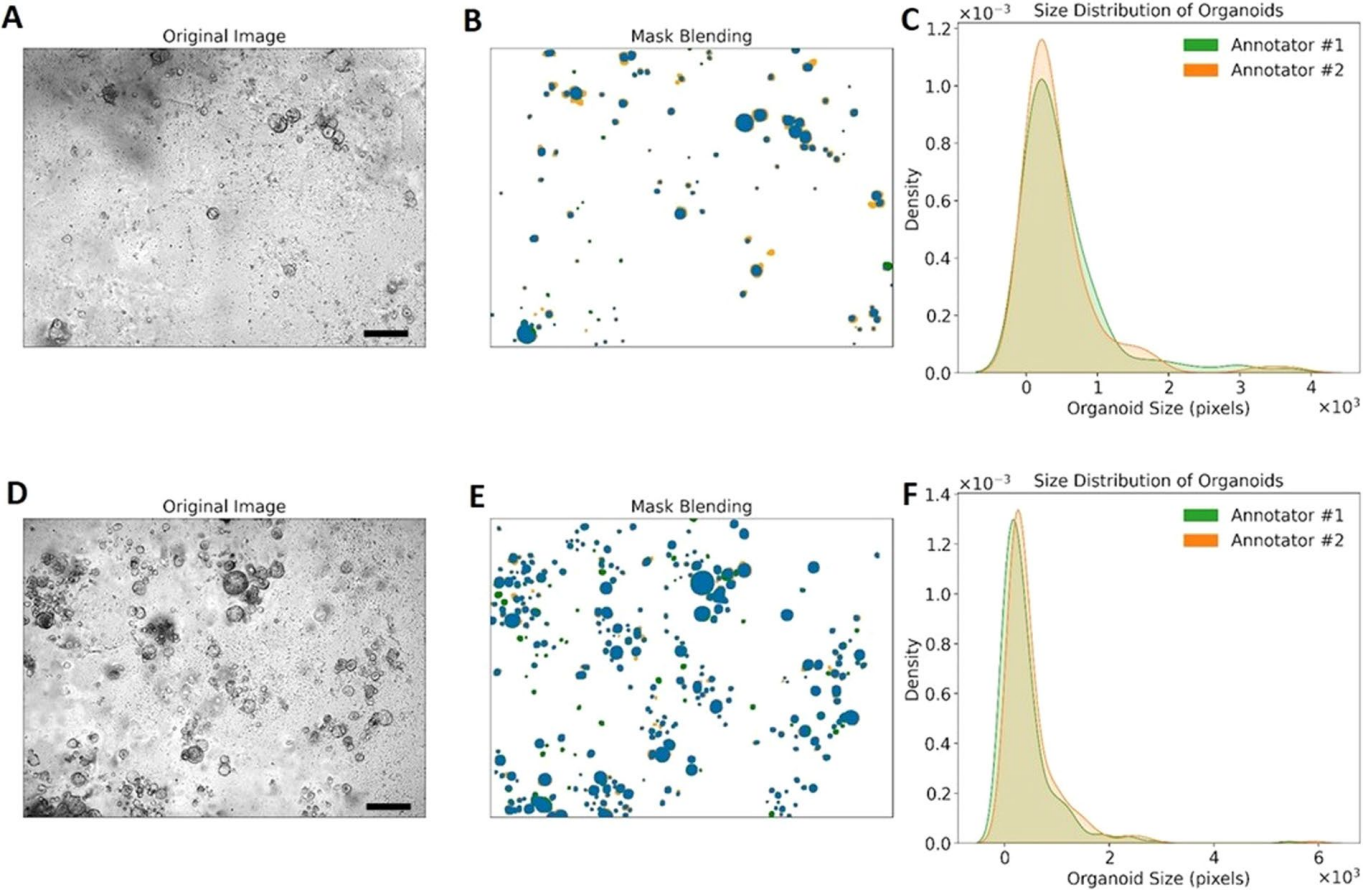
Inter-operator agreement analysis of a human and a mouse image of the OrganoIDNetData dataset. (**A**) reports a field of view acquired for PDAC organoids co-cultured with immune cells from “*Human_1_004h.tif*”, while (**D**) shows a field of view with organoids of “*Mouse_2_030h.tif*”. Scale bars: 400 *μm*. (**B**) and (**E**) display their respective masks, where the green color corresponds to the manual annotation by the first annotator, and the orange color represents the annotation by the second annotator. Common annotations are depicted in blue. (**C**) and (**F**) show the distributions of the two manual annotations, in terms of the areas of the recognized organoids.

Since a high-quality benchmark dataset requires accurate annotations, this section provides their analysis, manually performed by the two experts involved. We assessed this by computing an inter-annotator agreement metric. Images from both human (Fig. 3A) and mouse (Fig. 3D) PDAC organoids co-cultured with immune cells were independently annotated by the two annotators, resulting in two different masks for each image, respectively. Figure 3B and E report exemplary mask blendings, with green and orange indicating the annotations of the two experts and blue showing their overlap. For each image, we computed the objectwise percentage of organoids recognized by both annotators. For the dataset, we obtained an overall inter-annotator agreement of 21% ± 11%. This proves the complexity of the dataset and the need to consult more experts to achieve a good quality of the manual segmentation. Additionally, we analyzed the blended masks and feature distributions of the organoids recognized by the two annotators to understand the reasons underlying discrepancies between them. The distributions of the areas of the recognized PDAC organoids are similar, with the second annotator identifying a higher number of small structures than the first annotator for both human (Fig. 3C) and mouse samples (Fig. 3F). The blended masks of the two annotations reveal that regions with a higher density of organoids are the most challenging to segment, leading to a greater number of disagreements between the operators.

### StarDist and Cellpose-based segmentation

**Fig. 4:**
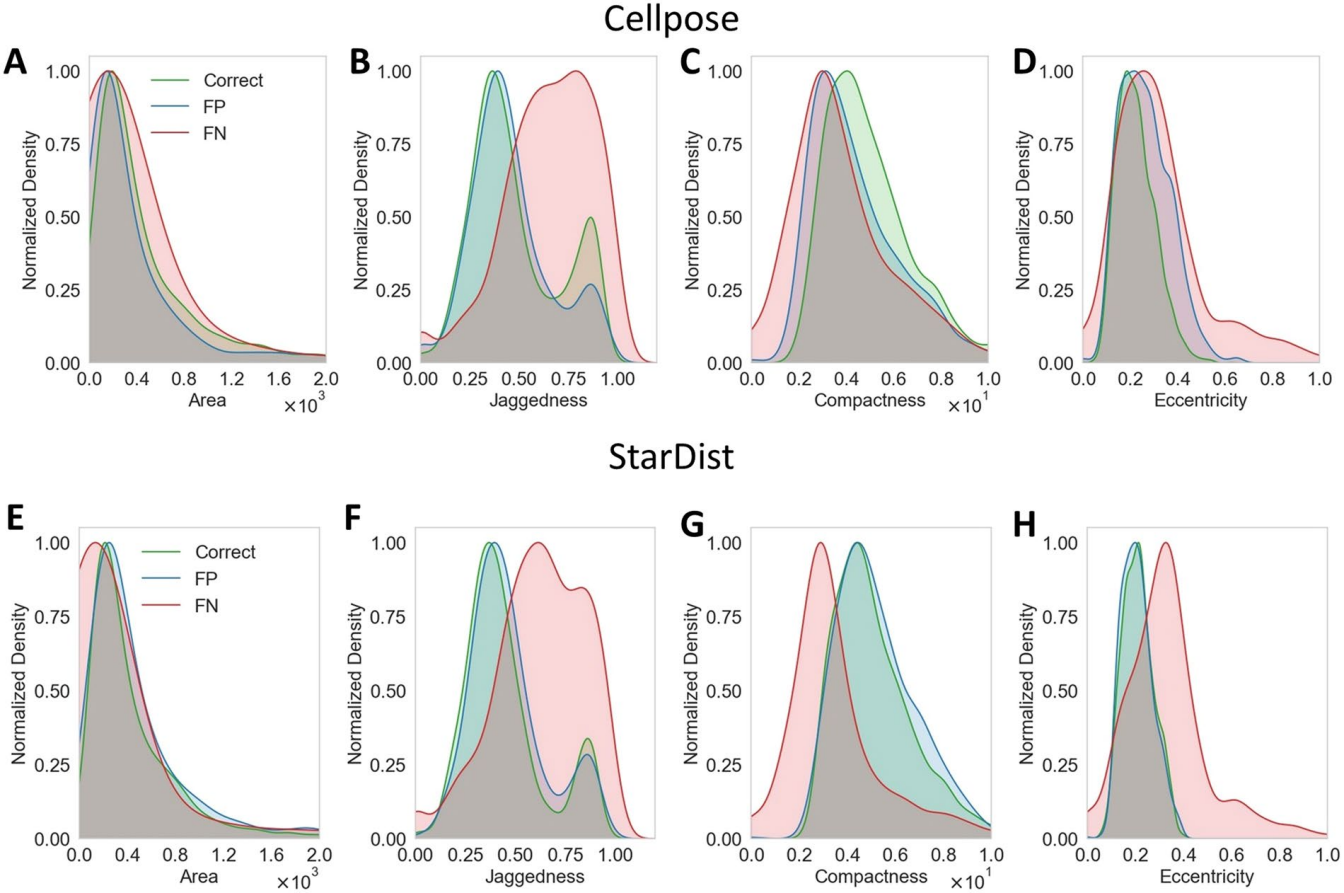
Comparative morphological analysis of the errors from Cellpose and StarDist segmentations. (**A**), (**B**), (**C**) and (**D**) display the distributions, normalized in the range [0, 1], of area, jaggedness, compactness and eccentricity, respectively, of the PDAC organoids co-coltured with immune cells correctly segmented by Cellpose (green), the False Positives (blue, contributing to the nFP metric) and the False Negatives (red, contributing to the nFN metric). (**E**), (**F**), (**G**) and (**H**) show the same distributions related to the outcome of StarDist. Area is expressed in pixels, while jaggedness, compactness and eccentricity are dimensionless.

Firstly, we used the corresponding subsets of the OrganoIDNetData dataset to train and test StarDist and Cellpose algorithms. For each image of the test dataset, both algorithms provided a segmentation mask, which was compared with the ground truth mask (i.e. the manual annotation) by computing different quantitative metrics able to assess the methods’ segmentation accuracy at object-level. The potential errors made by the algorithms, which require quantification, include the false identification of non-organoid structures as organoids (increasing the false positive rate) and the failing detection of organoids present in the image (increasing the false negative rate). Therefore, we computed the normalized false positive counts (nFP) and normalized false negative counts (nFN) metrics as the ratios between the number of false positives or negatives and the number of organoids identified in the ground truth. For each analyzed image, we obtained the standard deviation of each metric by partitioning the image into 6 regions of interest each measuring 800 × 800 pixels with a 400 pixel overlap in both directions, and computing the metric for each algorithm within these patches. This method ensured that high standard deviation reflected high variation of the metric across different regions within the image, indicating a less homogeneous field of view. Detailed results were reported in Table 1. Both StarDist and Cellpose demonstrated fairly good performances across human and murine organoid images. For both human and mouse images, Cellpose generally exhibited slightly lower nFP values compared to StarDist, suggesting fewer instances of non-organoid structures being erroneously identified as organoids. Moreover, this results indicated also a StarDist tendency to over-segment, particularly in more densely packed images. This is particularly evidenced for “*Test_Mouse_2_008h.tif*”, where the nFP values are 15.1% ± 6.7% and 11.6% ± 5.6% for StarDist and Cellpose, respectively. While both StarDist and Cellpose are effective for organoid segmentation in microscopy images, Cellpose demonstrated a slight advantage, particularly in more challenging samples such as murine tissues, owing to a high density of organoids present, compared to the human samples, where organoids are fewer and sparsely distributed. The high complexity of the OrganoIDNetData dataset relies also in the high PDAC organoid density of mouse images, where the nFN parameter dramatically increased for both segmentation methods. Discrepancy in organoids density is clearly visible also by comparing the field of view of human and mouse-derived PDAC organoids (for instance Fig. 3A and D, respectively). Additionally, we performed a detailed morphological analysis of the segmentation errors, whose comprehensive results are shown in Fig. 4. For each image listed in Table 1, we categorized the detected structures into three groups: correctly classified organoids, false positives (structures incorrectly identified as organoids by the segmentation algorithms), and false negatives (organoids missed by the segmentation algorithms). We evaluated the morphology of these groups by analyzing the distributions of four different metrics: organoids area (Fig. 4A and E for Cellpose and StarDist, respectively), jaggedness (Fig. 4B and F), compactness (Fig. 4C and G), and eccentricity (Fig. 4D and H). Jaggedness is defined as the ratio of the organoid perimeter to its area, providing a quantitative measure of the boundary complexity and organization of the organoids. Indeed, organoids with the same area but a more complex boundary shape is characterized by a higher value of the jaggedness parameter. Compactness is defined as the ratio of the organoid area to its perimeter, offering a quantitative assessment of how tightly the organoid occupies space. Eccentricity is defined as the ratio between the longest and the shortest axis of the ellipses, describing the degree of roundness of the organoid. To facilitate comparison among the distributions, for each graph we normalized the three curves related to the corrected regonized organoids, the false positives and the false negatives to the range [0, 1]. Both Cellpose and StarDist false positives displayed similar distributions, indicating that the overall morphology of these false positives closely resembles that of true organoids. Interestingly, PDAC organoids that were not recognized by either segmentation approach (increasing nFN) exhibited distinct overall morphological characteristics: they generally had a slightly lower area and compactness, and greater eccentricity and jaggedness, in comparison to both the false positives and the correctly recognized organoids. This wide variation in the morphological appearance among the organoids present in OrganoIDNetData underscores the complexity and challenge the dataset presents, even for robust segmentation algorithms.

**Table 1:** Quantitative assessment of Organoid Segmentation Algorithms.

Image	StarDist		Cellpose	
	nFP [%]	nFN [%]	nFP [%]	nFN [%]
*Test_Human_1_004h*	14.6 ± 7.3	14.6 ± 9.9	12.8 ± 9.1	8.5 ± 8.5
*Test_Human_1_008h*	16.7 ± 8.9	9.1 ± 9.7	16.9 ± 8.8	10.6 ± 9.9
*Test_Human_1_012h*	10.3 ± 6.0	12.5 ± 9.9	10.1 ± 7.0	11.3 ± 11.3
*Test_Human_1_016h*	18.5 ± 11.1	10.8 ± 7.2	18.5 ± 9.6	10.8 ± 7.7
*Test_Human_1_020h*	7.6 ± 7.6	16.4 ± 8.6	6.3 ± 2.9	19.2 ± 14.3
*Test_Mouse_2_000h*	12.3 ± 2.9	16.9 ± 2.3	11.2 ± 5.4	15.5 ± 2.8
*Test_Mouse_2_002h*	19.3 ± 4.0	16.9 ± 7.6	18.7 ± 6.7	19.0 ± 7.6
*Test_Mouse_2_004h*	19.5 ± 5.8	21.6 ± 4.7	18.0 ± 6.1	20.9 ± 7.2
*Test_Mouse_2_006h*	20.8 ± 11.0	21.6 ± 4.7	18.2 ± 10.0	22.6 ± 6.3
*Test_Mouse_2_008h*	15.1 ± 6.7	26.9 ± 4.2	11.6 ± 5.6	29.1 ± 6.7

This table presents the evaluation metrics for the performance of two organoid segmentation algorithms, StarDist and Cellpose, compared to ground truth masks. The metrics include the normalized False Positive counts (nFP) and normalized False Negative counts (nFN) calculated for each algorithm. The results showcase the accuracy and reliability of both algorithms in segmenting PDAC organoids co-cultured with immune cells from microscopy images at various time points. Lower values indicate better segmentation performance. The standard deviation of each metric was computed by splitting the image into 6 regions of interest of 800 × 800 pixels with 400 pixels overlap in both directions and computing the metric for each algorithm in these patches.

### Live cell imaging analysis

**Fig. 5:**
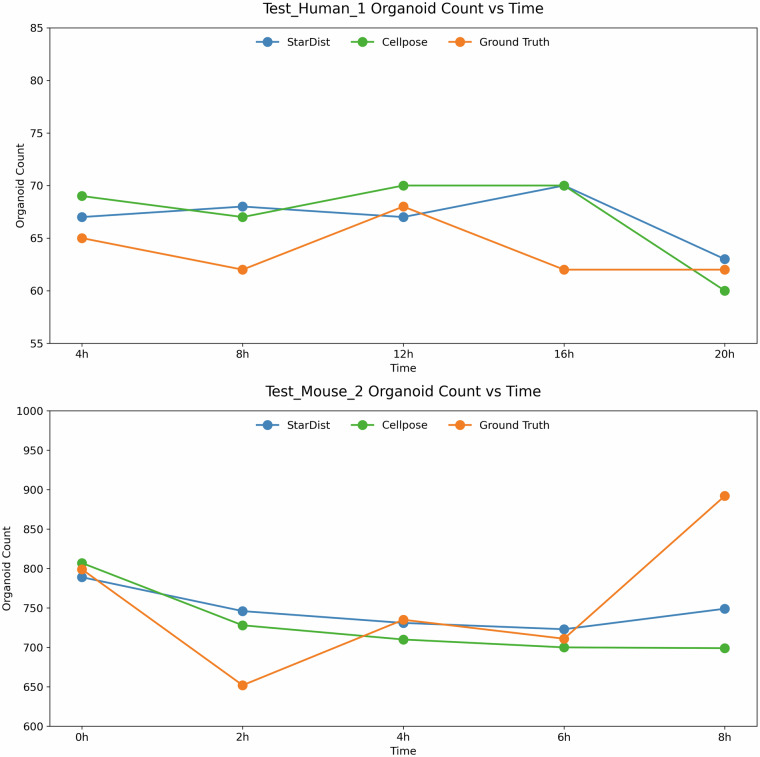
Comparative analysis between StarDist and Cellpose of two OrganoIDNetData time-series. (**A**) shows the organoid count obtained by Cellpose (green), and StarDist (blue), compared with the manual annotation outcome (orange) obtained during the analysis of the time-series involving the phase-contrast images acquired from the first human patient, recognized as “*Test_Human_1*” in the dataset. (**B**) shows the results obtained by performing the same analysis on the time-series involving the phase-contrast images acquired for a murine sample, specifically recognized in the dataset as “*Test_Mouse_2*”.

OrganoIDNetData can be also used to test the performance of novel algorithms in the analysis of time-series. By taking advantage of both StarDist and Cellpose, we analyzed two different time-series provided in the “*Test*” subfolder from the dataset, belonging to a human patient, specifically to a human sample (human 1), and to a murine sample (mouse 2). The overall results are provided in Fig. 5. The murine and human samples of the test dataset are characterized by a very different density of organoids, whose number spans roughly 1700 and 70 organoids per image, respectively. In both scenarios, StarDist and Cellpose reflect the trend characterizing the ground truth over time. Nevertheless, Cellpose typically identifies more organoids in the human samples compared to StarDist. The trends for mouse samples are more challenging to discern. In this context, StarDist tends to identify more organoids than Cellpose, primarily due to over-segmentation, as evidenced by its higher nFP values in Table 1. The high density of organoids in mouse sample images, particularly at the 8 hours time point, complicates the task for segmentation algorithms to accurately trace all organoids. This difficulty is indicated not only by the lower number of organoids identified at 8 hours but also by the significantly higher nFN values shown in Table 1 for both methods across all mouse samples. Furthermore, as already highlighted in^21^, the OrganoIDNetData dataset presents differences over time in general PDAC organoid features such as area, average count, and eccentricity. Additionally, pixel intensity allowed stratification of the organoids’ health status, with healthy organoids appearing bright and unhealthy ones appearing dark. Consequently, the biological complexity of the proposed time-lapsed configuration of the OrganoIDNetData dataset is a challenging and valuable opportunity to evaluate machine learning methods’ ability to simultaneously track organoid structural changes, providing a more comprehensive evaluation of segmentation algorithms’ performance and limitations on the simultaneous retrieval of morphology-related discrepancies over time.

### Evaluating morphological differences of human-derived organoids

**Fig. 6:**
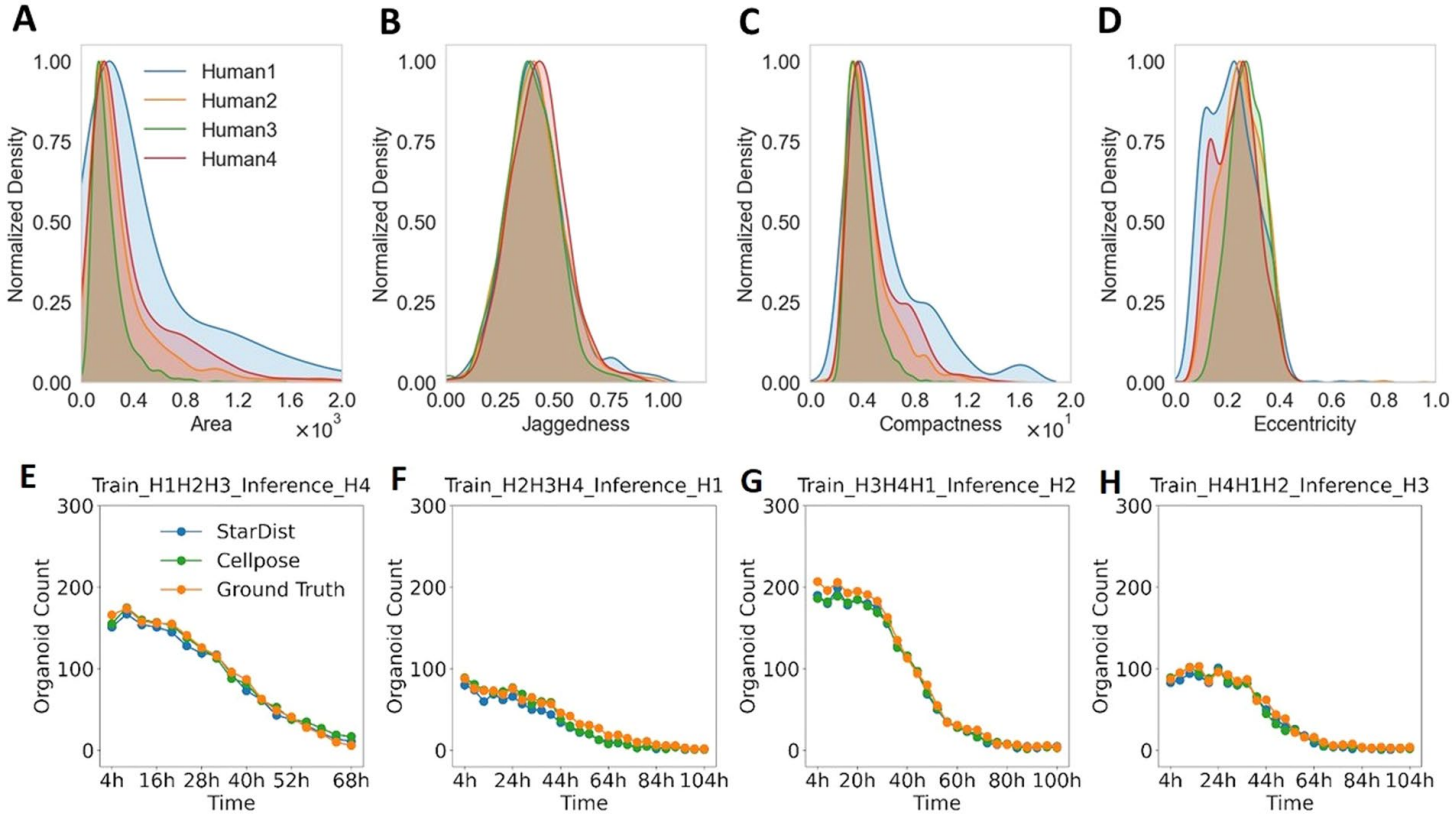
Morphological discrepancies among organoids derived from different patients. (**A**), (**B**), (**C**), and (**D**) show the distributions of area, jaggedness, compactness, and eccentricity, normalized in the range [0, 1], for organoids co-cultured with immune cells and derived from the four different human patients in the proposed dataset (blue, yellow, green, and red colors), respectively. Area is expressed in pixels, while jaggedness, compactness and eccentricity are dimensionless. (**E**) displays the organoid count over time for StarDist (blue) and Cellpose (green), compared with the ground truth (orange), when both algorithms are trained only on images from human 1, human 2, and human 3, and tested on an image representing organoids from human 4. The same analysis is shown in (**F**) for an image from human 1, where the training phase of both algorithms used images from human 2, human 3, and human 4 only. (**G**) presents the analysis with inference on human 2, trained on images from human 1, human 3, and human 4. (**H**) shows the analysis with inference on human 3, trained on images from human 1, human 2, and human 4.

Capturing the biological discrepancies occurring in real scenarios is another important property of the creation of a good benchmark dataset. For this reason, we conducted two analysis: 1) we analyzed the variations occurring in organoids derived from the four different patients in terms of morphological characteristics and 2) we investigated their effect during the training convergence and potential overfitting of AI-based segmentation methods StarDist and Cellpose. Comprehensive results of our investigation are reported in Fig. 6.

For the first analysis, we independently examined the morphology of PDAC organoids co-cultured with immune cells from the four human samples with the same four metrics we used during the comparative analysis of errors: the organoid area (Fig. 6A), jaggedness (Fig. 6B), compactness (Fig. 6C) and eccentricity (Fig. 6D). Overall, these histograms, normalized in the range [0 1] to enhance their visualization and comparison, indicate that the organoids from human 3 are the most homogeneous in terms of size, boundary complexity, and shape. In contrast, PDAC organoids from human 1 show the greatest variability across all measured characteristics. In particular, their area and compactness distributions are more clearly wider than the others. Human 2 and human 4 display intermediate levels of variability, with human 4 generally exhibiting a bit more spread than human 2. The jaggedness distributions are homogeneous for all the samples, centered in the range [0.3 0.4]: human 3 PDAC organoids are characterized by a slightly sharped distribution, while the distribution of human 1 is the only one to have a smooth secondary peak near 0.8.

For the second analysis, we trained both StarDist and Cellpose with images from three human samples and tested them on images of the fourth one, rotating through all possible combinations. This analysis demonstrates that the inferences for human 4 and human 1 (Fig. 6E and F) are slightly less accurate than those related to human 2 and human 3 (Fig. 6G and H), in particular for measurements around 28 and 44 hours from the start of the experiment.

This analysis underscores that the biological variability present in the proposed dataset is sufficient to pose a challenge also for robust and reliable state-of-the-art segmentation approaches, such as StarDist and Cellpose. Nevertheless, biological variability will be further improved by expanding our proposed collaborative dataset with images, acquired in other laboratories, of PDAC organoids derived from a higher number of different patients.

### Impact of immune cell enrichment in OrganoIDNetData dataset complexity

**Fig. 7:**
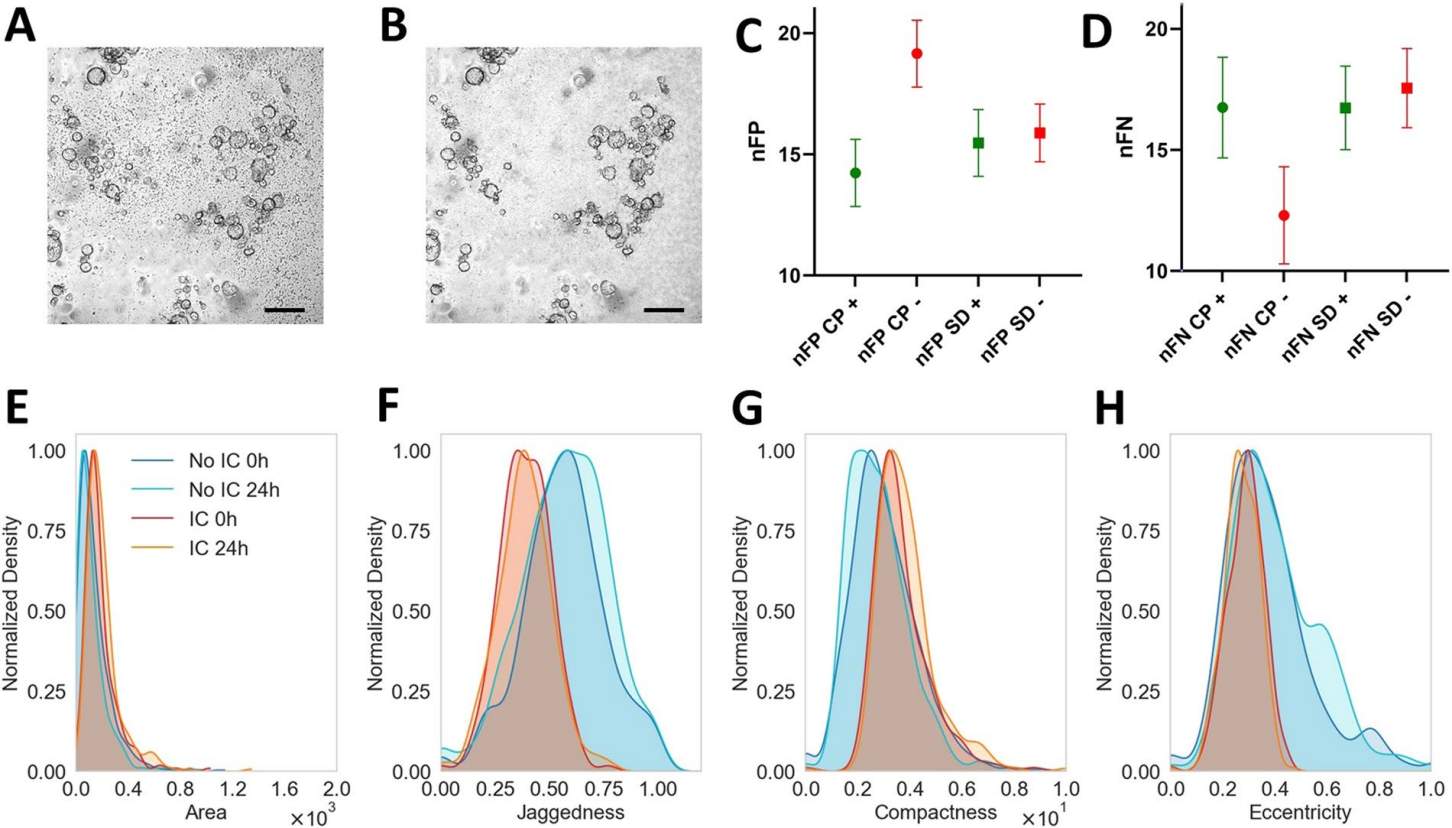
Computational and morphological analysis between organoids cultured with and without immune cells. (**A**) reports the dataset image, “*Mouse_2_012h_patch_4.tif*”, while (**B**) depicts its counterpart, where immune cells were artificially ablated. Scale bars: 320 *μm*. (**C**) shows the mean nFP obtained on the test images, with the corresponding standard deviations, by training Cellpose (CP, round dot) or StarDist (SD, squared dot) with the OrganoIDNetData training dataset, where immune cells are present (+, green color) and with the dataset where immune cells were artificially ablated (−, red color). (**D**) shows the same plot of (**C**) related to the mean nFN parameter. (**E**), (**F**), (**G**) and (**H**) display the distributions, normalized in the range [0, 1], of area, jaggedness, compactness and eccentricity, respectively, of the PDAC organoids of Human 3 in four different conditions: cultivated without immune cells at 0 and 24 hours from the beginning of the experiment (blue and cyan distributions, respectively) and co-coltured with immune cells at 0 and 24 hours from the beginning of the experiment (red and orange distributions, respectively). The area is expressed in pixels, while jaggedness, compactness and eccentricity are dimensionless.

We further demonstrated that the presence of immune cells significantly contributes to the higher complexity in segmenting our dataset, compared to state-of-the-art datasets that lack immune cells. This was achieved through two different experiments, the results of which are shown in Fig. 7. In the first experiment, we compared the segmentation performance of Cellpose and StarDist on a test dataset using the nFP and nFN metrics. Both algorithms were trained on the OrganoIDNetData training dataset, but the training images were preprocessed to perform an in silico ablation of the immune cells. Unlike creating a completely new image dataset of organoids cultivated without immune cells, which could introduce discrepancies due to various factors such as image acquisition conditions, the artificial ablation of immune cells preserved all other characteristics of the training dataset. Consequently, any performance variation must be attributed solely to the absence of the virtually deleted structures. Since immune cells appear as small black dots in the images, artificial ablation was performed on all training set images by selecting all structures characterized by a small area (under 100 pixels), a small ratio between area and perimeter (less than 0.1), and high contrast compared to the neighboring background (the mean intensity of the structure had to be at least 30% less than the nearby background). Each ablated pixel was replaced with the mean grayscale intensity of the nearest 64 pixels, excluding ablated pixels. An exemplary image from the dataset is shown in Fig. 7A, while its processed counterpart after virtual ablation is depicted in Fig. 7B. The mean results for the nFP parameter (Fig. 7C) show that both Cellpose and StarDist exhibited lower performance when trained with the dataset without immune cells (14.2% ± 1.4% with immune cells and 19.2% ± 1.4% without immune cells, for Cellpose, and for 15.5% ± 1.4% and 15.9% ± 1.1%, respectively, for StarDist). This indicates an increased tendency for the algorithms to oversegment, particularly for Cellpose, which tends to recognize immune cells as organoids. Indeed, the ablation prevented the algorithms from learning about these structures from the manual annotations, which avoided immune cells, leading to issues when these structures suddenly appeared in the test dataset images. Regarding the nFN parameter (Fig. 7D), while StarDist showed slightly worse performance, Cellpose exhibited better performance, recognizing a higher number of true organoids. This is a direct consequence of the oversegmentation discussed in Fig. 7C: since Cellpose segments more structures, it is more likely to recognize additional organoids, though its reliability is compromised by misidentifying many immune cells as organoids. The reduced performance of Cellpose and StarDist on the OrganoIDNetData test dataset, when trained with images lacking immune cells, demonstrates that the presence of immune cells directly contributes to a more complex segmentation environment, arising not only from a biological perspective but also from the difficulty of segmentation. The second experiment focused on the morphological analysis or organoids, investigating if potential discrepancies in organoids shape cultivated with or without immune cells could have an impact in the reduced segmentation accuracy. To achieve this, we maximally reduced the interpatient biological heterogeneity by considering PDAC organoids derived from human 3 only. Specifically, we computed the human 3 organoids distributions for area (Fig. 7E), jaggedness (Fig. 7F), compactness (Fig. 7G) and eccentricity (Fig. 7H) for four different conditions: 1) organoids cultivated without immune cells at 0 hours (i.e. immediately after the beginning of the experiment), 2) the same organoids after 24 hours from the beginning of the experiment, 3) organoids co-cultured with immune cells at 0 hours, 4) the same organoids after 24 hours from the beginning of the experiment. The distributions show clearly that the two populations exhibit slightly different morphologies: in presence of immune cells, organoids appeared a bit bigger, with more uniform boundaries. Moreover, their compactness increased. Eccentricity distributions are centered around 0.3 for all the distributions, but the organoids cultivated without immune cells showed a more heterogeneous roundness. Furthermore, at this time point, it is interesting that organoids without immune cells showed a slightly higher jaggedness and a decreased compactness, while in presence of immune cells, the compactness slightly increased, together with an overall slight reduction of the eccentricity parameter and a more homogeneous jaggedness distribution. Furthermore, after 24 hours, no substantial differences were detected in the distributions of the areas for both populations. This analysis emphasized that slightly morphological alterations are also caused by the presence of immune cells, leading to a more complex segmentation task when the algorithms analyze a dataset composed by images acquired in both conditions.

## Usage Notes

In our knowledge, OrganoIDNetData represents the first imaging dataset involving organoids co-cultured with immune cells. It is distinguished by meticulous manual annotations for every field of view. Its dimension, in terms of number of images, variety and number of organoids, is similar or higher than other datasets used to validate novel segmentation protocols^14,16,18^. The substantial variability in organoid density across the dataset images makes it a particularly challenging benchmark for the evaluation of segmentation protocols. The manual annotation, executed by experts, suggests an opportunity for enrichment with annotations contributed by more scientists. This collaborative effort would enhance the ground truth quality, mitigating potential subjectivity arising from a single expert. Up to now, the OrganoIDNetData dataset, whose images are consistently acquired within the same laboratory under similar conditions, is limited to murine and human PDAC samples, co-cultured with immune cells, and focuses on providing a challenging and common benchmark for object detection and segmentation AI-driven algorithms, as the proposed Cellpose and StarDist.

Inspired by initiatives undertaken for tumor samples, as The Cancer Genome Atlas^28^ or The Cancer Image Archive^29^, OrganoIDNetData marks an initial stride towards establishing a more complex collaborative dataset. Such a dataset will provide an interesting data collection for many different AI algorithms, from multiclass classification to unsupervised learning-based protocols. Indeed, it would encompass the interpatient heterogeneity^30^, featuring both images and numerical data, spanning a broad spectrum of patient derived organoids from different tumors. The collaborative dataset will encompass many different conditions for each type of organoids, providing images and data of organoids cultivated with and without immune cells and undergoing different experimental treatments. The provided scripts, leveraging Cellpose and StarDist-based analysis are properly commented to facilitate user customization. Although they are not optimized to guarantee the best performance, their parameter settings are a reliable starting point for users during their preliminary analysis. Then, for instance, users could implement on-the-fly data augmentation to further improve the algorithms’ segmentation accuracy and facilitate convergence during the training phase. Moreover, each code is designed for compatibility with other datasets, provided they adhere to the same organizational structure as the proposed one.

## Data Availability

The codes used in this article are available for free in the dedicated GitHub public repositories https://github.com/ajinkya-kulkarni/PyOrganoIDNet and https://github.com/ajinkya-kulkarni/PyBlendPatches, under the CC-By License v4.0. The pre-trained model weights and the dataset presented are freely accessible from the corresponding Zenodo repository^26^.
